# Functional Alteration of a Dimeric Insecticidal Lectin to a Monomeric Antifungal Protein Correlated to Its Oligomeric Status

**DOI:** 10.1371/journal.pone.0018593

**Published:** 2011-04-07

**Authors:** Nilanjana Banerjee, Subhadipa Sengupta, Amit Roy, Prithwi Ghosh, Kalipada Das, Sampa Das

**Affiliations:** 1 Division of Plant Biology, Bose Institute, Kolkata, India; 2 Department of Chemistry, Bose Institute, Kolkata, India; St. Georges University of London, United Kingdom

## Abstract

**Background:**

*Allium sativum* leaf agglutinin (ASAL) is a 25-kDa homodimeric, insecticidal, mannose binding lectin whose subunits are assembled by the C-terminal exchange process. An attempt was made to convert dimeric ASAL into a monomeric form to correlate the relevance of quaternary association of subunits and their functional specificity. Using SWISS-MODEL program a stable monomer was designed by altering five amino acid residues near the C-terminus of ASAL.

**Methodology/Principal Findings:**

By introduction of 5 site-specific mutations (-DNSNN-), a β turn was incorporated between the 11^th^ and 12^th^ β strands of subunits of ASAL, resulting in a stable monomeric mutant ASAL (mASAL). mASAL was cloned and subsequently purified from a pMAL-c2X system. CD spectroscopic analysis confirmed the conservation of secondary structure in mASAL. Mannose binding assay confirmed that molecular mannose binds efficiently to both mASAL and ASAL. In contrast to ASAL, the hemagglutination activity of purified mASAL against rabbit erythrocytes was lost. An artificial diet bioassay of *Lipaphis erysimi* with mASAL displayed an insignificant level of insecticidal activity compared to ASAL. Fascinatingly, mASAL exhibited strong antifungal activity against the pathogenic fungi *Fusarium oxysporum, Rhizoctonia solani* and *Alternaria brassicicola* in a disc diffusion assay. A propidium iodide uptake assay suggested that the inhibitory activity of mASAL might be associated with the alteration of the membrane permeability of the fungus. Furthermore, a ligand blot assay of the membrane subproteome of *R. solani* with mASAL detected a glycoprotein receptor having interaction with mASAL.

**Conclusions/Significance:**

Conversion of ASAL into a stable monomer resulted in antifungal activity. From an evolutionary aspect, these data implied that variable quaternary organization of lectins might be the outcome of defense-related adaptations to diverse situations in plants. Incorporation of mASAL into agronomically-important crops could be an alternative method to protect them from dramatic yield losses from pathogenic fungi in an effective manner.

## Introduction

Mannose binding monocot plant lectins are inherently capable of defending the organism from predators and infectious pathogens. They possess one or more carbohydrate binding domains that bind reversibly to specific mono- or oligosaccharides [1]. These carbohydrate binding domains are diverse in structure and, therefore, vary in binding specificity. Based on the available sequence and structural information, the majority of all known plant lectins have been subdivided into seven structurally and evolutionarily related groups [2]. Among them, “monocot mannose binding lectin” is a well-conserved superfamily composed primarily of bulb lectins found in the plant families of Amaryllidaceae, Alliaceae, Orchidaceae, Araceae, Liliaceae and Bromeliaceae. Despite strong sequence conservation, they typically vary in the tertiary structure and quaternary organization that provides the greatest insight into their functionality in a biological system [3]–[4]. Incidentally, some lectins are monomeric proteins (Gastrodianin), some are stable at the dimeric level (Garlic lectin), and in some cases, the subunits associate to form tetramers (Snowdrop lectin). Although not a universal characteristic, it has been observed that the biological roles of lectins vary considerably depending upon oligomerization features [5]. Dimeric lectin has specific antagonistic effects towards insects and monomers are inhibitors of fungal growth whereas tetramers exhibit an anti-retroviral property. For instance, snowdrop lectin, or GNA (*Galanthus nivalis*, Amaryllidaceae), is a tetramer known to be a potent inhibitor of HIV and other retroviruses, due to its ability to bind gp120 [6], the major glycoprotein exposed on the surface of an HIV envelope. In contrast, garlic lectin, which has no detectable antiretroviral activity, can bind to proteins glycosylated by high mannose such as invertase and alliinase with very high affinity [7]–[8] and has been reported to have a controlling ability against a varied range of homopteran insect attacks [9]–[11]. The structural basis for these differences in binding specificity as well as the variations in quaternary association in monocot mannose binding lectins with regards to their function is, therefore, of great interest. Hence, an attempt has been made to address the correlation between various quaternary organizations of garlic lectin (ASAL) with their functionalities and possible evolutionary implications.

The potent insecticidal protein ASAL (Accession No. AY866499), which is isolated from the garlic leaf, is a naturally occurring homodimer wherein each subunit contains three potential mannose binding motifs made up of five amino acid residues: Gln, Asp, Asn, Val and Tyr (QDNVY). These 5 residues comprising the polar surface of the binding pockets are completely conserved in all of the subdomains throughout this lectin super family [12]. The primary understanding of the structure of this super family is from analysis of tetrameric GNA and dimeric *Allium sativum* bulb lectin. In all studied structures [5], the two subunits have a β-prism II fold structure, similar to that of the snowdrop lectin, comprising three anti-parallel four-stranded β-sheets arranged as a 12-stranded β-barrel, with an approximate internal 3-fold symmetry; they assemble into a tightly-bound dimer by exchanging their C terminal β-strands to form a hybrid β-sheet [13]. This mode, frequently referred to as the C-terminal exchange, provides a large buried area on the subunit/subunit interface through which a stable dimer is established. At this point, the question that remains to be answered is which key factors serve as the driving forces for this quaternary association. Identification of these factors necessitates a detailed understanding of the process of dimerization as well as the development of a stable monomeric form that illuminates the evolutionary relationship between various oligomers.

Henceforth, in our present study, we have designed experiments to unravel which secondary structural elements of ASAL are responsible for dimerization. A computer-based homology modeling program was adopted to design a stable monomer from the dimeric form of ASAL that suggested insertion and replacement of five amino acid residues –DNSNN- in the dimeric protein. Accordingly, five site-specific mutations were subjected at amino acids 101–105 of ASAL. Basically, a β-turn was incorporated between the 11^th^ and 12^th^ β-strand of the protomer, leading to the formation of a stable monomer. The novel monomeric protein was then expressed and purified using a pMAL-c2X expression vector and characterized using biophysical and biochemical tools. Conservation of the secondary structure of mASAL was authenticated by CD spectroscopic analysis. The mutant ASAL was tested for insecticidal activity against homopteran pests as a comparison with ASAL. The biological activity of the mutant lectin was also analyzed against fungal pathogens that cause diseases in various crops. An insight into the mode of action of the monomeric protein towards fungal pathogens was achieved through the use of a propidium iodide uptake assay and a ligand blot assay. Thus, our analyses advance understanding of the correlation between the quaternary structure and function of the mannose binding lectin ASAL and its mutant form mASAL. Based on the aforementioned findings, it can be suggested that the newly designed mASAL could serve as a potential candidate for incorporation into agronomically important crop plants to protect them from fungal attack.

## Materials and Methods

### Design of monomeric form of ASAL

To identify and characterize the critical residues involved in the dimerization of ASAL, multiple sequence alignment was performed using ClustalW2 [14] with the representative members of monocot mannose binding lectin superfamily (ACAL, AML, ACL, GAFP, GNA). A computer simulated monomeric model was generated using the program SWISS-MODEL [15]–[17] to visualize mutated amino acid residues with respect to ASAL in order to identify the structural determinants for oligomerization.

### Stepwise PCR amplification for mutagenesis in ASAL

The nucleotide sequence of mutant protein (mASAL) was generated using the Quick Change Site Directed Mutagenesis Kit (Stratagene, USA). Mutagenesis experiments were performed by multiple rounds of PCR amplification according to the manufacturers' protocol. Three sets of mutagenic primers (Table 1) were used for the purpose of stepwise PCR amplification. In three consecutive PCR reactions, one insertion and four replacement mutations were performed. The reaction conditions were 1 cycle of 30 sec at 95°C followed by 20 cycles of 30 sec at 95°C, 1 min at 55°C and 1 min at 68°C, followed by a final extension step of 10 min at 72°C in a MyCycler (Bio Rad, Hercules, CA, USA). Stepwise PCR amplification was performed using a native ASAL sequence initially as a template, and the intermediate amplified product was used as the template for the following rounds of PCR. After purification from an agarose gel using a Qiagen gel purification system (GmbH, Hilden, Germany), the product was cloned, and nucleotide sequencing of the insert was performed using an automated ABI Prism 377 Sequencer (Applied Biosystems) at the sequencing facility of the Bose Institute, Centenary campus, India.

**Table 1:** List of primers used in stepwise PCR amplification for replacement and insertion mutation in ASAL.

PCR number	Primer combination	Primer sequence
1st PCR	Primer 1	5′AGCTGGATCCATGGCCAGGAACCTACTGACGAACGGTGA 3′
	Primer 2	5′ATTATCGTAAATGACAACGTTC 3′
2nd PCR	Primer 1	5′AGCTGGATCCATGGCCAGGAACCTACTGACGAACGGTGA 3′
	Primer 3	5′AGACCAAATCGCATTATTAGA 3′
3rd PCR	Primer 1	5′AGCTGGATCCATGGCCAGGAACCTACTGACGAACGGTGA 3′
	Primer 4	5′AACCTAGGTACCAGTAGACCAAATCGC 3′

### Expression and purification of mASAL

Mutant ASAL (mASAL) coding a gene carrying the replacement G98D and an insertion of Asn99, Ser100, Asn101, and Asn102 was further cloned at the BamH1 site in the polylinker region between the *mal E* gene and the *lacZα* gene, downstream of the gene encoding MBP in the pMAL-c2X expression vector using the pMAL protein fusion and purification system kit (New England Biolab, Ma USA), and the positive clone was designated pMAL-mASAL. The clone was expressed efficiently in an *E. coli* BL21 cell line (Invitrogen, California, USA). Expression and purification steps for the fused MBP-mASAL were performed using the kit according to the manufacturers' protocol. All purification steps were carried out at 4°C.

### Affinity chromatography

Following IPTG induction of the recombinant cell line, the cells were sonicated. The sonicated cell suspension was centrifuged, and the supernatant was diluted to a 1∶5 ratio by volume with a column buffer (20 mM Tris-HCl, 200 mM NaCl, 1 mM EDTA pH 7.4). The diluted extract was loaded on an amylose resin at a flow rate of 1 ml/min. The column was then washed with 12 column volumes of the same buffer. The fusion protein was eluted with a column buffer containing 10-mM maltose. Ten to twenty fractions 3 ml in volume were collected. The fusion protein began to be eluted within the first 5 fractions and was easily detected by UV absorbance at 280 nm. The protein-containing fractions were pooled and extensively dialyzed against 20-mM TBS (pH 7.4).

### Cleavage of the fusion protein

The fusion protein was digested with Factor Xa (New England Biolabs, Ma USA) at 25°C using the pMAL protein fusion and purification system kit (New England Biolab, Ma USA) according to the manufacturer's protocol. Factor Xa cleaved the protein at a particular cleavage site (Ile-Glu/Asp-Gly-Arg↓) of the fusion protein.

### Removal of maltose by hydroxyapatite column chromatography and domain separation by rebinding MBP to amylase

Hydroxyapatite resin was swollen in 20 mM TBS (pH 7.4) and poured in the column. A fusion protein cleavage mixture was loaded onto the column, and then the column was washed with the same buffer. Elution was performed using 0.5 M TBS (pH 7.4), and fractions were collected as 2-ml aliquots. Again, affinity chromatography was performed by loading the hydroxyapatite-eluted fractions onto the amylose column. The flow through was collected as 5-ml fractions, and the concentration of the protein was analyzed using the Bradford method [18]. The protein purified in this manner was extensively dialyzed against TBS (pH 7.4).

### Size determination and Western blot analysis of the purified protein

The monomeric form and purity of the protein was confirmed by 15% native as well as SDS-PAGE analysis according to the method proposed by Laemmli [19]. The separated proteins were electro-blotted to a positively-charged Hybond-C membrane (Amersham Biosciences, Buckinghamshire, UK). The membrane was blocked with 5% (w/v) BSA solution in 20-mM TBS and incubated for 1 h with an anti-MBP antibody (New England Biolab) and an anti-ASAL polyclonal primary antibody raised in rabbit at 1∶10,000 dilution. After washing, the membrane was further challenged with diluted (1∶20,000) anti-rabbit IgG-horse radish peroxidase (HRP) conjugate (Sigma, St. Louis, USA) as a secondary antibody for 1 h. The membrane was washed twice with 20-mM TBS, and the western blot was developed in Kodak film using an ECL western blot kit (Amersham Biosciences, Buckinghamshire, UK).

### Analytical Gel filtration analysis

Oligomerization status of mutated ASAL (mASAL) was further confirmed by the following experiment using native ASAL protein as control. Molecular size of the native ASAL and mASAL were measured using a Biosep-SEC-S-2000 column of Phenomenex (size: 300×7.8 mm). The two columns were calibrated with low and high protein molecular weight markers, comprising bovine serum albumin (M_r_ 67.0), ovalbumin (M_r_ 43.0), cytochrome C (M_r_ 29.0), ribonuclease A (M_r_ 13.7), and aprotinin (M_r_ 6.0). The elution buffer used for both columns was 0.1-M sodium phosphate (pH 6.8). Samples were eluted at a flow rate of 2 ml/min.

### Secondary structure determinations

Conservation of the secondary structure was followed by circular dichroism spectroscopic analysis using a CD Spectrometer (JASCO Corp. J-815CD spectropolarimeter with a temp. controller). CD spectra of ASAL and mASAL were recorded over a wavelength range of 200 to 260 nm. Measurements of the sample were taken in quartz cuvettes with a 0.1-cm pathlength at a temperature of 25°C. The protein concentrations were approximately 0.2 mg/ml in TBS (pH 7.4). Spectra were obtained as an average of 10 scans and measured in TBS (pH 7.4) and on a degree ellipticity (q) scale. Analysis of CD spectra in terms of the secondary structure content was performed using CDNN software.

### Dissociation Constant (*K_d_*) of mannose-bound complex using fluorescence spectroscopy

mASAL and ASAL were separately incubated at 25°C on a Hitachi F-7000 spectrofluorimeter using a Sigma cuvette (volume: 1 ml; pathlength: 1 cm). The solutions were titrated with 10 mM mannose in phosphate buffer (pH 7.2) by adding a small aliquot at a time. Following each addition, the solution was stirred using a magnetic stirrer for 1 min and the fluorescence emission spectrum was recorded between 300 and 400 nm using 295 nm as the excitation wavelength. The excitation and emission band passes were each 5 nm. D-glucose and N-acetyl glucosamine (NAG) were also used for a ligand binding experiment in a similar manner. The equation for single-site ligand binding measured through changes in the spectroscopic signal [20] is given by 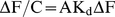
(1)where ΔF represents the increase or decrease in fluorescence intensity at a given concentration of the ligand, *K*_d_ is the dissociation constant, and *A* =  *K*_d_Δ *F*max. We used equation (1) to calculate the dissociation constant (*K*_d_) for binding of mannose, glucose and NAG to mASAL and ASAL.

### Hemagglutination assay

Blood was collected from the rabbit into a syringe pre-filled with 500-µl 0.9% NaCl solution and the blood was immediately transferred to a culture tube pre-filled with 0.9% saline. Subsequently, the erythrocyte solution was prepared by repeated washing with 0.9% saline and spun at 2500 g for 10 minutes at 4°C. After each cycle, the supernatant was carefully removed. The erythrocytes obtained in this manner were found to be free from leucocytes and cell debris. The erythrocytes were resuspended in 0.9% saline as a 2% (3.5×10^8^ cells/ml) cell suspension. Hemagglutination activity of the purified mASAL and /or ASAL was assayed in a 96-well microtiter U-plate according to a 2-fold serial dilution procedure (50, 25, 12.5, 6.25 µg/ml). The samples were diluted with PBS (137 mM NaCl, 2.7 mM KCl, 10 mM Na_2_HPO_4_, 1.8 mM KH_2_PO_4_; pH 7.4) at which point 50 µl of protein from each dilution was mixed with 50 µl of a 2% suspension of rabbit erythrocytes. The microtiter U-plate was kept for 1 hour at 37°C, and then hemagglutination was observed with the unaided eye [**10**].

### Insect bioassay

Insect bioassays were conducted using an artificial diet [9]–[11]. Twenty second instar nymphs of *Lipaphis erysimi* were used in multiple sets. Polycarbonate petriplates (perforated for air passage at the bottom) were used as a bioassay cage. Nymphs were released into the plates, and the upper edges of the plates were covered with fully stretched parafilm. A modified synthetic diet mixture of 200 µl, supplemented with different concentrations of ASAL and mASAL (5, 10, 15, 20, 25 µg/ml), was dispensed on the stretched parafilm membrane. Another Parafilm membrane was stretched onto this in order to form a pouch. For control plates, 20-mM TrisCl (pH 7.4) was added to the artificial diet instead of experimental proteins. For another set of control experiments, aphids were fed with only 20 mM TrisCl (pH 7.4) without the artificial diet. Each set of experiments for each dose was repeated four times. Data regarding the numbers of nymphs surviving were recorded at 12-h intervals for 72 h. The LC_50_ values for the toxicity against mustard aphids were determined by Probit analysis [21] (**Table 2**).

**Table 2:** Dissociation constants of mASAL and ASAL towards mannose, D-glucose and NAG.

Protein	Ligand	Dissociation constant (K_d_) in µM
mASAL	Mannose	0.12
	D-glucose	0.16
	NAG	0.11
ASAL	Mannose	0.06
	D-glucose	0.14
	NAG	0.3

### Disc Diffusion assay against fungal species

Antifungal activity was determined under sterile conditions using a hyphal extension-inhibition assay as described by Roberts and Selitrennikoff [22]. Fungal mycelia were harvested from actively growing fungal plates and placed in the center of Petri plates (100 mm×15 mm) containing 10 ml of potato dextrose agar (PDA) used for maintenance of the fungus under test. The antifungal activity was observed by incubating mASAL (5, 10, 15, 20 µg) in sterile paper discs (0.65 cm in diameter) placed at a distance of 0.5 cm from the rim of fungal mycelia of *Fusarium oxysporum, Alternaria brassicicola, Fusarium lycopersici* and *Rhizoctonia solani.* A solution of 10 mM Phosphate buffer (pH 7.4) and/or native ASAL (20 µg) were used in place of mASAL as a control. These plates were sealed with parafilm and incubated at 28°C to allow for mycelial growth to a diameter of 3 cm. Growth inhibition was assessed in the form of crescents of inhibition after incubation for 48 hrs at 28°C. Each set of experiments was performed in triplicate.

### Propidium iodide uptake assay on pathogenic fungi

The fungal membrane permeabilization allowing interaction with ASAL/mASAL was assessed using a Propidium Iodide (PI) uptake assay [23]–[24] conducted on *F. oxysporum, R. solani* and *A. brassicicola*. The permeabilization assay consisted of 200 µl of half-strength PDB containing fungal spores (1000 spores/ml) treated with mASAL, α-D mannose saturated mASAL and ASAL independently at concentrations of 4 µg for each fungal isolate. Control samples contained untreated fungal isolates. Fungal strains were incubated at 25°C for 40 hours. Each experiment for each fungal strain was performed in triplicate. After incubation, the samples were washed with 1xPBS (pH 7.4) and stained for 10 min in PI staining solution (25 µg/ml PI in PBS). Stained samples were washed twice with 1× PBS (pH 7.4) and viewed under an Axioscope Carl Zeiss inverted fluorescent microscope. Images were captured with the AxioCam ICc3 digital camera and AxioVision imaging software system (Carl Zeiss Micro Imaging, GmbH, Germany). An identical assay was performed with *Xanthomonas oryzae* Bxo 43 to investigate whether mASAL is capable of membrane damage, leading to PI uptake in organisms other than fungus. The presence of fluorescence is indicative of a compromised fungal/bacterial membrane.

### Immunolocalization Assay of mASAL and ASAL in *R. solani*

To visualize direct binding of mASAL compared to ASAL, to the fungal membrane, an immunolocalization assay was performed using an anti-ASAL antibody as the primary antibody. Fungal strains were first incubated separately with mASAL, ASAL (4 µg) and PBS buffer (pH 7.4; as a negative control). Another negative control set was generated by omitting the primary antibody in the reaction condition. After incubation at 25°C for 40 hours, the samples were washed with 1× PBS (pH 7.4) and further incubated in 2% buffered glycine followed by washing with 1× PBST at room temperature. Overnight blocking of the samples was carried out in 2% buffered BSA at 4°C. After thorough washing in 1× PBS (pH 7.4), the samples were incubated with an anti-ASAL antibody (1∶8000) for 2 hours at room temperature, followed by washing with 1× PBST and incubation with an anti-rabbit IgG-FITC conjugate (1∶20000) (Sigma-Aldrich, USA) for 1 hour at room temperature. Bound proteins were detected by anti-rabbit IgG-FITC conjugated secondary antibodies. Each experiment was performed in triplicate. The slides were examined using an Axioscope Carl Zeiss inverted fluorescent microscope. Images were captured with the AxioCam ICc3 digital camera and the AxioVision imaging software system (Carl Zeiss Micro Imaging, GmbH, Germany). The presence of green fluorescence is indicative of bound protein on fungal membrane.

### Ligand blot assay

Fungal membrane enriched fraction or membrane subprotome of *R. solani* were extracted by the protocol described by Meijer et al. 2006 [25] and Asif et al. 2006 [26], respectively. Extracted proteins were precipitated via a TCA/acetone precipitation method [27]. Then the protein was resolubilized in 1% SDS in 50 mM Tris-HCl (pH 7.0) and quantified using the Bradford method [18]. Approximately 10 µg of fungal membrane proteins were resolved in 12% SDS-PAGE and later on transferred electrophoretically to a Hybond-C membrane (Amersham Biosciences) in a Hoeffer submerged electroblot apparatus. The membrane was blocked with 5% nonfat milk solution in 1× TBST (pH 7.2) at 37°C for 1 hour. The membrane was then washed with TBST (pH 7.2) and incubated with 20 µg of mASAL for 2 hours at 37°C. After washing with TBST, the blotted membrane was further incubated for 1 hour at 37°C with the anti-*ASAL* polyclonal antibody at 1∶10,000 dilutions and the anti-rabbit IgG- horse radish peroxidase (HRP) conjugate as the secondary antibody at 1∶20,000 dilutions. Bound secondary antibodies were detected by enhanced chemiluminescence using the western lightning^ TM^ chemiluminescence reagent plus (PerkinElmer Life Science).

### Glycoprotein-Specific Staining

Glycospecific staining of the sub proteome of *R. solani* was performed with a Pro-Q Emerald 300 glycoprotein stain kit (Invitrogen, CA, USA) according to the manufacturer's protocol. The same gel was post-stained with SYPRO Ruby staining gel to stain the glycosylated as well as the non-glycosylated protein.

### Deglycosylation, mannose inhibition and subsequent Ligand blot analysis

The total membrane protein enriched fraction of *R. solani* was examined via a deglycosylation experiment using an N-Glycosidase F deglycosylation kit (Roche Applied Science, Mannheim, Germany) according to the protocol described in the kit's manual. Approximately 10 µg of membrane subproteome was taken, and 20 µl of denaturation solution (supplied with the kit) was added to it and then incubated in boiling water for 3 minutes. After allowing the solution to return to room temperature, reaction buffer (supplied with the kit) was added to the tube and incubated at room temperature for 15 minutes. Recombinant PNGase F was added to the mixture and incubated at 37°C for three hours. The deglycosylated sample was boiled with a SDS-PAGE sample buffer and subsequently resolved in 12% SDS-PAGE. Finally, a ligand blot was performed as mentioned previously and subsequently documented. The effect of α-D-mannose on mASAL-receptor interaction was monitored. mASAL pre-saturated with 1 M α-D-mannose was used to interact with the subproteome and subsequently probed with an anti-ASAL antibody in the ligand blot assay. Finally, the membrane was developed accordingly to the procedure described above [9], [28].

## Results

### Design of the monomeric mutant form of ASAL

**Figure 1:**
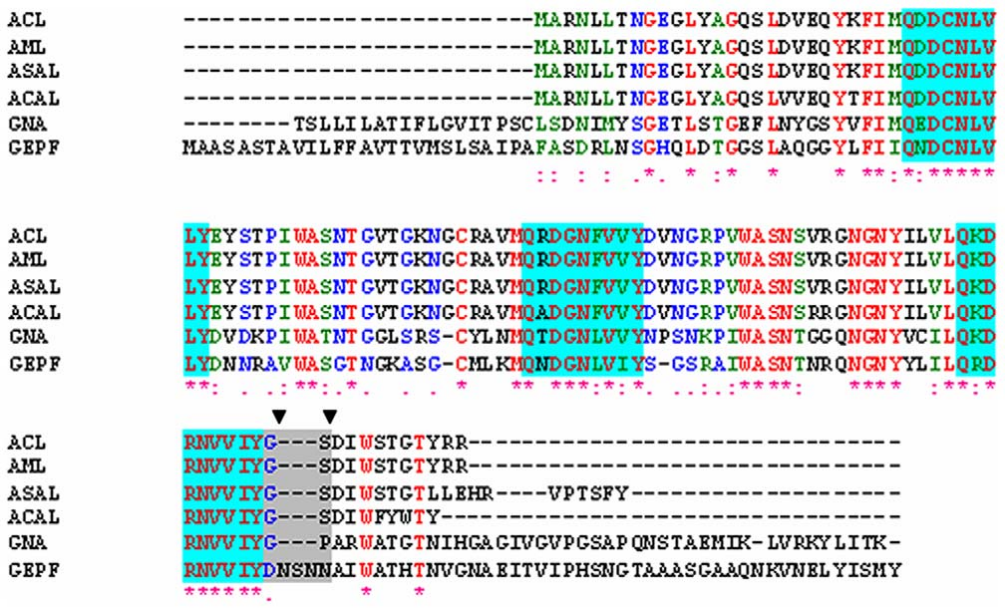
Alignment analysis of the deduced amino acid sequence of ASAL with closely related mannose binding homologues. Sequence alignment among ASAL, *Amorphophallus paeonifolius* lectin (ACL), *Arum maculatum* lectin (AML), *Allium cepa* lectin (ACAL), *Galanthus nivalis* agglutinin (GNA) and Gastrodianin (GAFP). All identities and similarities are indicated by (*) and (:). Blue blocks and the grey box represent all mannose binding domains and the stretch of amino acids that differ in the monomer, respectively. The two residues at positions 98 and 99 (GNA numbering) are shown with two down triangles, where a trans-peptide bond is present in monomers instead of the cis-peptide bond found in all oligomers.

**Figure 2:**
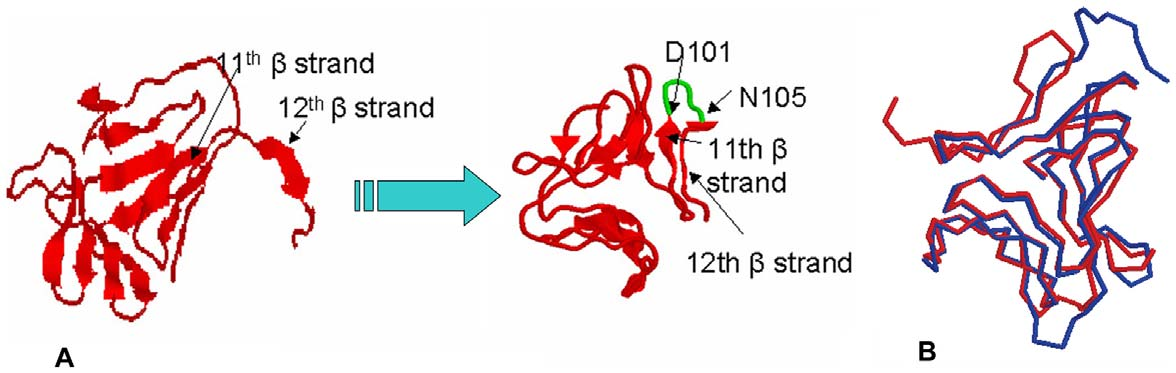
Comparison between the homology models of ASAL and mASAL. (A) The homology model of ASAL to mASAL, showing that after incorporation of the loop between the 11^th^ and 12^th^ β-strand, the flanking C-terminal peptide folds back towards the central axis and thereby maintains the overall β-prism II fold. (**B**) Superimposition of mASAL (red) and the counterpart of ASAL (blue). The inflection point for the radical shift appears at position 98 (GNA numbering), from which point the C-terminal peptide moves in a completely different direction. In ASAL, the C-terminal peptide protrudes from the central axis of the molecule, whereas in mASAL, the 12^th^ β-strand is folded to form a homogeneous β-sheet.

On the basis of multiple alignments of sequences of ASAL-related lectins (Figure 1) and homological modeling (Figure 2) supported by preliminary crystallographic data [5], a stretch of five amino acids were identified to be responsible for the generation of a stable monomeric form. Dimeric ASAL resembles the general β-prism II fold consisting of three sub-domains, I, II, and III, each of which forms a flat four stranded β-sheet. A total of 12 β-strands are arranged to form a β-barrel where a pseudo three-fold axis is located in the center and relates the three faces of the triangular prism. A number of nonpolar side chains point to the centre of the β-barrel forming a hydrophobic core stabilized by a network of strong Van Der Waals interactions. Among these, Trp 41, Trp74 and Trp103, located in subdomains III, II and I, respectively, are conserved residues. They are kept invariant through all the sequences studied thus far (**Figure 1**) and are believed to play a crucial role in stabilizing the overall structure of the protein. Structural analysis suggested that in ASAL, dimerization is maintained by the hydrophobic interaction between the 12^th^ β-strands of two neighboring subunits (stable dimer) that are associated tightly. Thus, it would be logical to design monomeric ASAL by disrupting the subunit association at the dimeric interface. Therefore, we constructed a plausible model of monomeric ASAL by the incorporation of a β-turn just prior to the C-terminal 12^th^ β-strand of ASAL. The stretch of five amino acids Asp98, Asn99, Ser100, Asn101, and Asn102 form a β-hairpin instead of a loop between the 11^th^ and 12^th^ β-strands (**Figure 2A**). All of these amino acids have the propensity to be located in the β-turn. Interestingly, substitution of Glycine with Asp98 contributed to the monomerization process. Glycine is the most favored residue at position 97 (GNA numbering) in all reported oligomers because it forms a *cis*-peptide bond with the next residue (threonine in case of native ASAL). As a result, the remainder of the residues towards the C-terminus are kept in such an orientation that they can interact with the C-terminal residues of another subunit while aspartic acid of mASAL in place of glycine ensures the formation of a *trans* peptide bond with the next Asn 99 residue. This five-residue motif belonging to a 3∶5 β-hairpin with an internal β-bulge renders the following C-terminal segment reversible in order to contact the flanking peptide chain (11^th^ β-strand) of the same subdomain to establish an intramolecular homogeneous 4-stranded β-sheet. The backbone of the β-hairpin is well established by a local H-bond network mediated by hydrophilic side chains. From structural point of view, the presence of such a β-hairpin arising from residue replacement and insertion in the sequence of ASAL the peptide beyond mutation has to shift radically from its original position and orientation in the oligomeric state (**Figure 2B**). Such a rearrangement of the C-terminal peptide appeared to bring about a radical decrease or even a complete disappearance of the buried surface at the interface between two molecules, and thereby contributes greatly to the stabilization of the monomeric state.

### Mutagenesis, expression and purification of stable monomeric protein (mASAL)

**Figure 3:**
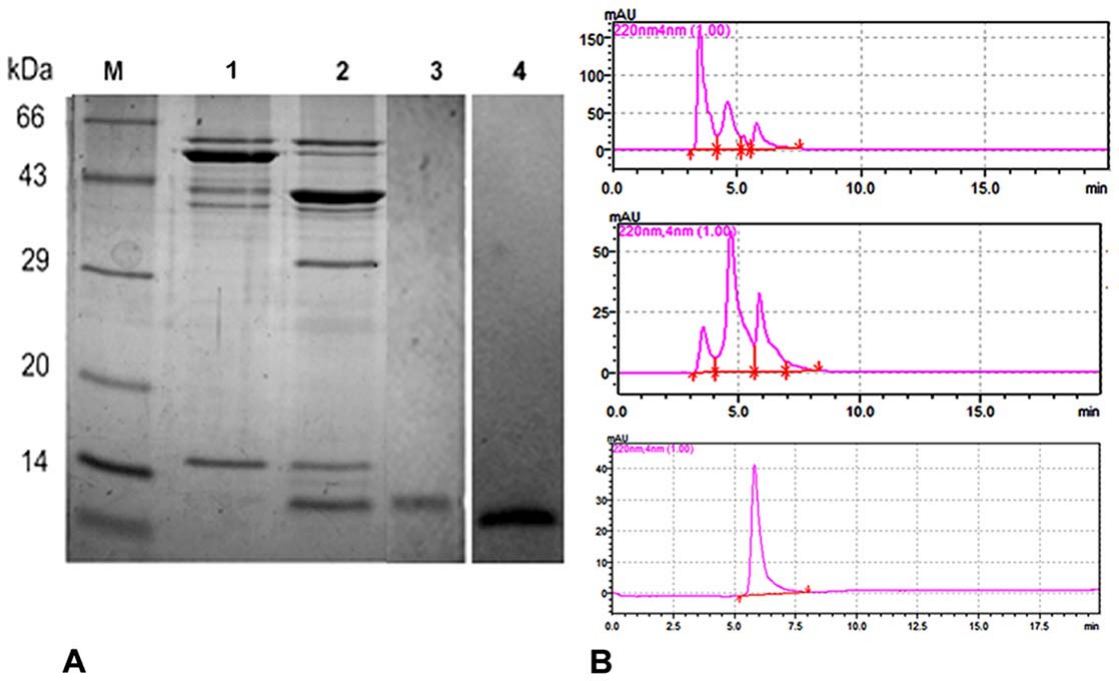
Elution profile and expression analysis of mASAL. (**A**) Expression and purification of mASAL in 15% SDS-PAGE analysis; lane 1 represents MBP fusion protein; lane 2 represents fusion protein digested with Factor Xa; lane 3 represents hydroxyapatite column purified mASAL; lane 4 represents Western blotting of purified mASAL against an anti-ASAL antibody showing a band at 12.5 kDa; lane M represents a standard protein molecular weight marker. (B) The corresponding elution profiles from size exclusion chromatography results of the mutant protein from a Biosep-SEC-S-2000 column of Phenomenex at the flow rate 2 ml/min. The figure shows fused mASAL with MBP (pick at 3.5 min), MBP and mASAL after factor Xa digestion (picks at 4.8 min and 6.15 min, respectively) and pure mASAL (pick at 6.1 min).

In the present work, five mutations were introduced between the 11^th^ and the 12^th^ ß-strands of wild type dimeric ASAL. The first mutation was achieved by replacing glycine at position 98 with aspartic acid. Next, the other four residues -N-S-N-N- were efficiently introduced via two consecutive PCR amplification steps. The mutant ASAL coding gene was cloned using a pMAL-c2X expression vector and the resulting protein was expressed in a BL21 cell line of *E. coli* under IPTG induction. The appearance of a ∼56 kDa band in SDS-PAGE indicated the purities of the expressed protein after 4 hours of IPTG induction (**Figure 3A**). After affinity chromatography and 30 hours of Factor Xa digestion, mASAL was purified. The purified product was analyzed in 15% SDS PAGE, which detected distinct bands at approximately 43 kDa and 12.5 kDa (**Figure 3A**). Western blotting **(** **Figure 3A** **, lane 4)** with monoclonal anti MBP antibody and anti ASAL polyclonal antibody confirmed the purified mASAL production.

### Gel filtration chromatography as well as native PAGE analysis of purified mASAL

**Figure 4:**
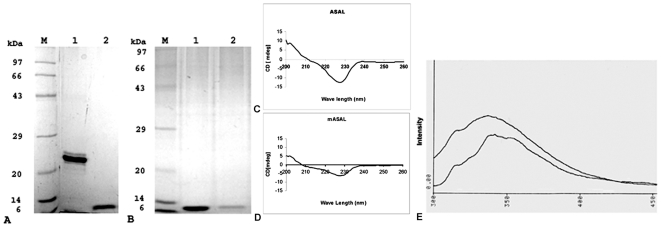
Size determination, Molecular characterization and secondary structure determination of mASAL and ASAL. (**A**) Dimeric ASAL and monomeric mASAL were resolved in 15% native gel; Lane 1 represents purified ASAL showing a band in the 25-kDa region, lane 2 represents purified monomeric mASAL with band of size 12.5 kDa. (**B**) Proteins were resolved in 15% SDS-PAGE, lane 1 and lane 2 represent dimeric ASAL and mASAL, respectively. Both show bands in the 12.5-kDa region. Lane M represents the Standard protein molecular weight marker. Conservation of the secondary structure of (**C**) ASAL was determined by comparing the circular dichroism spectra with (**D**) mASAL. CD spectra were recorded over a wave length range of 200 to 260 nm. Spectra were obtained as an average of 10 scans and measured in PBS (pH 7.4) at a temperature of 25°C. The protein concentrations were approximately 0.2 mg/ml in PBS (pH 7.4). (**E**) Fluorescence spectra of Native ASAL and mASAL, where protein concentrations were 0.15 mg/ml. Excitation was performed at 295 nm and emission was scanned in the wavelength range of 300 to 400 nm. The slight red shift in the fluorescence spectra of mASAL observed was due to a change in the sub-domain organization after C-terminal self assembly in the monomeric form.

On the elution profile of the Biosep-SEC-S-2000 column, the peak of the protein appeared at an elution volume of 9.2 ml (expected time: 4.6 minutes), corresponding to an apparent molecular weight of approximately 25 kDa in the case of native ASAL (Data not shown). On the elution profile of the Biosep-SEC-S-2000 column, however, the mutant protein was eluted at a volume of roughly 12.3 ml (expected time for elution: 6.15 minutes), which is consistent with the monomeric molecular size of the recombinant protein. The elution peaks of recombinant fused proteins are shown in Figure 3B, which clearly indicates the purity of the protein. The results of gel filtration analysis also indicated the change in the quaternary state of the dimer to a monomeric form of native ASAL caused by mutation. The data were further validated by comparing ASAL and mASAL in native PAGE as well as SDS- PAGE analysis (**Figures 4A, B**). In native PAGE, a distinct band at 25 kDa and a band at approximately 12.5 kDa were resolved in case of ASAL and mASAL, respectively (**Figure 4A** **)**. This data confirmed the conversion of mASAL as stable monomer.

### Conservation of the secondary structure in the monomer

The CD spectra of ASAL and mASAL were characterized by monitoring the minima at approximately 228 nm with a negative positive crossover at 211 nm and 208 nm, respectively **(** **Figures 4** **C, D)**. The overall secondary structural component of the respective proteins was estimated as an alpha helix of 6.4% and 7.4%, an anti-parallel beta sheet of 20.5% and 19.3%, a parallel beta sheet of 19.4% and 18.9%, a beta turn of 22.3% and 21.8%, with the remainder of the structures contributed by random coils. Small variations observed in the structural organization were primarily due to changes in sub-domain organization following C terminal self-assembly in a monomeric form. The data were further validated through fluorescent spectroscopic analysis of ASAL and mASAL (**Figure 4** **E**). Due to mutation, a slight change in conformation might have occurred in mASAL which actually caused the red-shift of λ-max from 332 nm to 340 nm (approximate).

### Binding affinity of mannose to mASAL

Because native ASAL belongs to the monocot mannose binding lectin superfamily, the binding of mASAL and ASAL to mannose was ensured. Previous studies have established the fact that ASAL binds to oligomannosides with a preference for α 1, 2 linked mannose residues [8], [29]. Man9GlcNAc2Asn, which carries several α 1, 2 linked mannose residues was the best mannooligosachharide ligand in this respect (binding affinity K_a_ = 1.2×10^6^ M^−1^ at 25°C).

**Figure 5:**
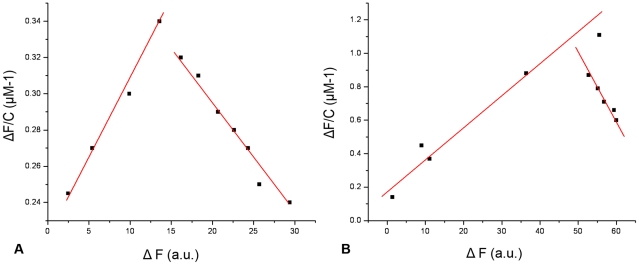
Determination of dissociation constant (K_d_) for the interaction of mannose with mASAL and ASAL. The protein concentration used was 0.15 mg/ml. The dissociation constant was calculated from the linear plot of ΔF/C against ΔF, where ΔF represents the increase or decrease in fluorescence intensity at a given concentration of mannose. (A) and (B) represent the binding of mannose with mASAL and ASAL where the dissociation constants were 0.12 µM and 0.06 µM, respectively.

When mASAL was titrated with mannose, there was a distinct difference in absorbance, indicating the binding of mASAL to mannose. The dissociation constant (K_d_) of mASAL was calculated to be 0.12 µM. For a single mannose moiety, the calculated dissociation constant of ASAL for mannose was 0.06 µM. The values of dissociation constants of mASAL and ASAL towards mannose indicate that ASAL binds to a single mannose molecule much more efficiently than does mASAL. This also suggests that mASAL is intended to be structurally stable and biologically active as it can bind mannose even at the monomeric level. This also points to the fact that in spite of the introduction of 5 charged residues, all of the three putative mannose binding domains remain intact. The conserved side chains present in the binding pocket of mASAL coincide well with those of ASAL and GNA. This similarity in the geometry of the binding pockets confirms the strong preference of mASAL for the axial hydroxyl group at position 2 in the ligand, which is a common property among other members of the same family. The change of slope in the binding profile may suggest a possible conformational change of ASAL and mASAL (**Figure 5**). For other sugar residues, such as D-glucose, the binding affinity of ASAL and mASAL appeared to be almost identical as indicated by the dissociation constants. In the case of NAG, however, the binding affinity of mASAL was found to be higher than that of ASAL. The dissociation constants of mASAL and ASAL for mannose, D-glucose and NAG are shown in Table 2. Moreover, upon addition of urea (up to 8 M), mASAL follows the same unfolding and refolding pathway as is observed in case of native ASAL (data not shown). All of these findings suggest that the 5-residue motif acts as the structural switch for dimer to stable monomer conversion in addition to maintaining the integrity of the monomeric structure so that mASAL can bind to mannose and remain biologically active.

### Insect bioassay and hemagglutination assay

**Figure 6:**
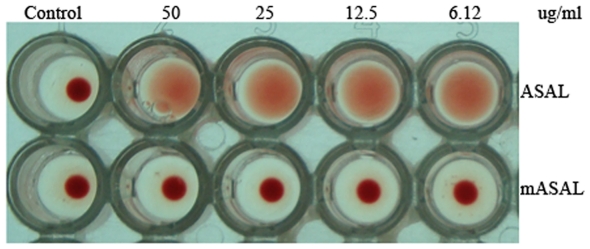
Hemagglutination assay of rabbit erythrocyte with different doses of ASAL and mASAL. Microtiter wells represent an agglutination pattern of 100 µl of 1% rabbit erythrocytes with various doses of ASAL and mASAL. Well 1 in vertical rows 1 and 2 represent negative controls containing PBS buffer (pH 7.4). Wells 2–5 in vertical rows one and rows two represent ASAL and mASAL with different doses (50 µg/ml to 6.12 µg/ml), respectively.

From insect bioassay experiments, it was evident that the effect of ASAL is more potent as a toxin than is mASAL on *Lipaphis erysimi.* The LC_50_ value of ASAL against the aforementioned insect pest is 20.7 µg/ml, which is almost four times lower than that of mASAL (LC_50_ value: 78.98 µg/ml) (**Table 3**) as calculated from mortality data. The hemagglutination assay of mASAL, when compared to ASAL, exhibited a loss of the agglutination property in mASAL (**Figure 6**). In control wells and wells containing mASAL, a tight button of red cells indicative of negative reaction was observed. In contrast, agglutinated cells form a carpet over the wells containing ASAL. These results suggested that, in mASAL, the insecticidal property of ASAL was substantially decreased and the agglutination property was completely lost.

**Table 3:** Comparative susceptibility of *Lipaphis erysimi* to ASAL and mASAL.

Sample	LC_50_ value (µg/ml)	Fiducial Limit 95%	Regression Equation	SE of Slope	λ 2 Value	d.f
						
ASAL	20.7	18.52–23.93	y = 2.91+1.58 x	0.21	0.58	3
						
mASAL	78.98	60.38–118.14	y = 1.77+1.60 x	0.38	0.24	3

### Assay for antifungal activity

**Figure 7:**
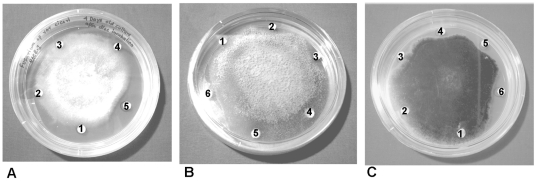
*In vitro* disc diffusion assay of mASAL. *In vitro* antifungal activity of mASAL against (**A**) *Fusarium oxysporum* f.sp. *cicero*, (**B**) *Fusarium lycopersici*, and (**C**) *Alternaria brassicicola.* mASAL (5, 10, 15 µg) was applied to the filter discs numbered 3–5 of panel **A** and 3–6 of panel **B** and panel **C** (with 5,10,15,20 µg of mASAL). Discs 1 and 2 of panels **A, B** and **C** are 10-mM sodium phosphate buffer and purified ASAL (20 µg), respectively.

Mutated ASAL had an antifungal effect *in vitro* against a number of plant pathogenic fungi. We compared the antifungal effect of mASAL on the hyphal growth of *Fusarium oxysporum varciceri* (Figure 7A), *Fusarium lycopersici* (Figure 7B), *Alternaria brassicicola* (Figure 7C) *and Rhizoctonia solani* (data not shown). Phosphate buffer was used as negative control. The effect of ASAL was also evaluated on the same fungal plate. After 48 hrs, a crescent-shaped inhibition zone appeared around all of the discs with the exception of that corresponding to the phosphate buffer and native ASAL. All three phytopathogenic fungi demonstrated a similar effect. Significant inhibitory activity was found at a protein concentration of 15–20 µg (**Figure 7**).

### Propidium iodide uptake assay

**Figure 8:**
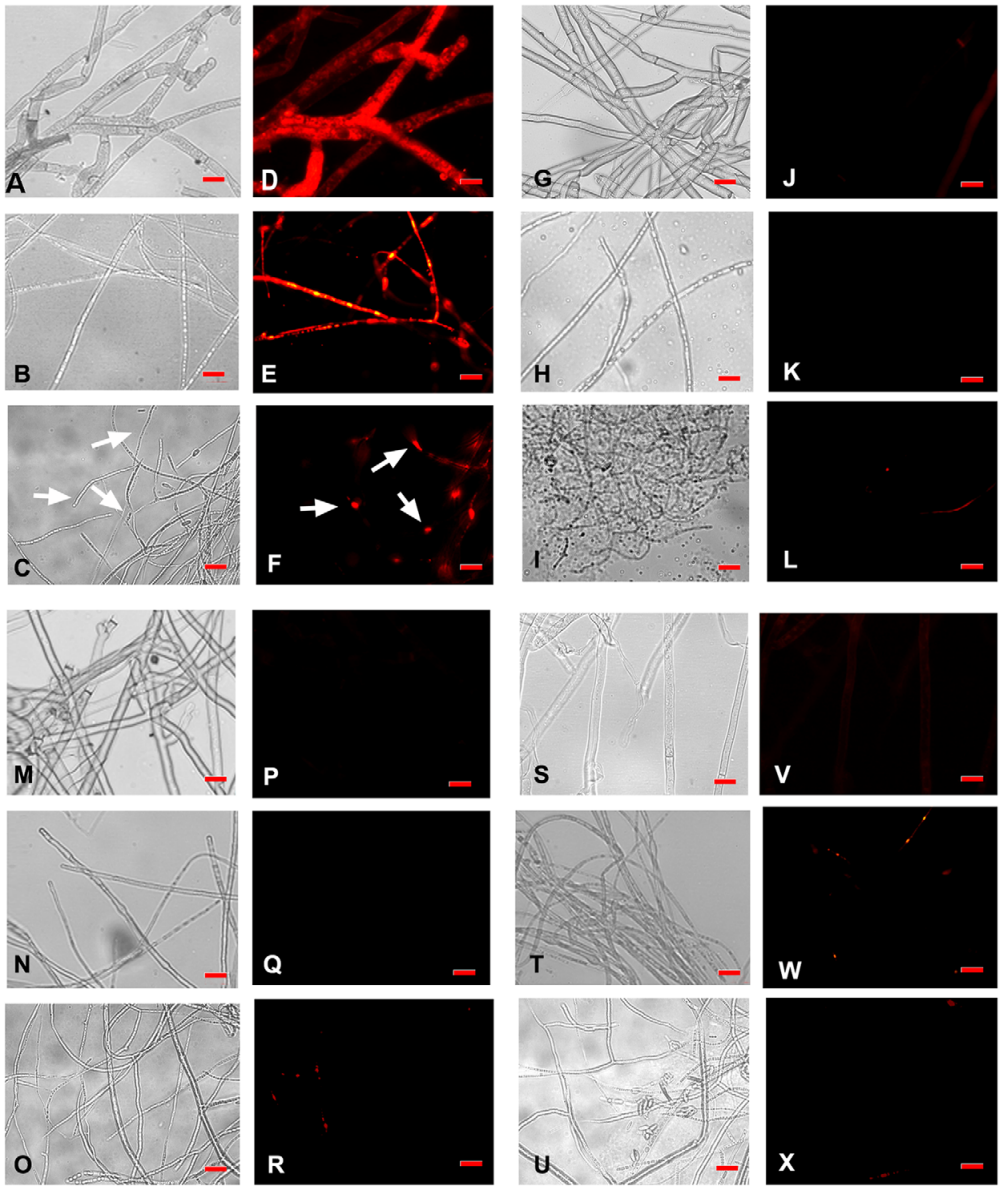
Fluorescent microscopic analysis of a propidium iodide uptake assay. (**A, B, C**) and (**D, E, F**) are respective light microscope images and fluorescent images of mASAL treated *R. solani*, *F. oxysporum* and *A. brassicicola*. (**G, H, I**) and (**J, K, L**) are light microscope images and fluorescent images of mASAL pre-saturated with excess α-D mannose treated *R solani*, *F. oxysporum* and *A. brassicicola*, respectively. (**M, N, O**) and (**P, Q, R**) are light microscope images and fluorescent images of ASAL-treated *R solani*, *F. oxysporum* and *A. brassicicola*, respectively. (**S, T, U**) and (**V, W, X**) are respective light microscope images and fluorescent images of untreated *R solani*, *F. oxysporum* and *A. brassicicola*. Fungi were grown for 40 hours in the presence of mASAL and/or ASAL at peptide concentrations of 4 µg. Untreated fungi were taken as control. Afterwards, fungal hyphae were stained with Propidium iodide for 10 min, washed with 1× PBS, and subjected to fluorescent microscopic analysis. Bar = 15 µm. Images were captured with the AxioCam ICc3 digital camera and AxioVision imaging software system (Carl Zeiss Micro Imaging, GmbH, Germany).

A propidium iodide treatment used in combination with fluorescent microscopy revealed high levels of fluorescence in the mASAL-treated samples (**Figure 8** **D, E, F**) when compared to mASAL pre-saturated with α-D mannose (**Figures 8** **J, K, L**) and/or ASAL-treated samples (**Figures 8** **P, Q, R**) as well as the untreated fungi that showed no fluorescence (**Figures 8** **V, W, X**). Fluorescence was observed throughout the affected hyphae in *Fusarium, Rhizoctonia and Alternaria* strains, while the effect was increased in *Rhizoctonia*; in the case of *A. brassicicola,* fluorescence was detected only at some parts of mASAL-treated hyphae as shown in **Figure 8** **F** (indicated by white arrow). The data indicated that mASAL exhibited its antifungal effect by disrupting fungal membrane integrity, allowing for the uptake of propidium iodide. In contrast, fungal membranes remained intact when they were treated with ASAL and/or mASAL that was pre-saturated with excess mannose and therefore excluded propidium iodide. These data point towards the lectin activity of mASAL. The same assay, when executed on bacteria with mASAL, displayed no fluorescence, indicating the fungi-specific nature of mASAL (**Figure S1**).

### Immunolocalization assay using anti ASAL antibody

**Figure 9:**
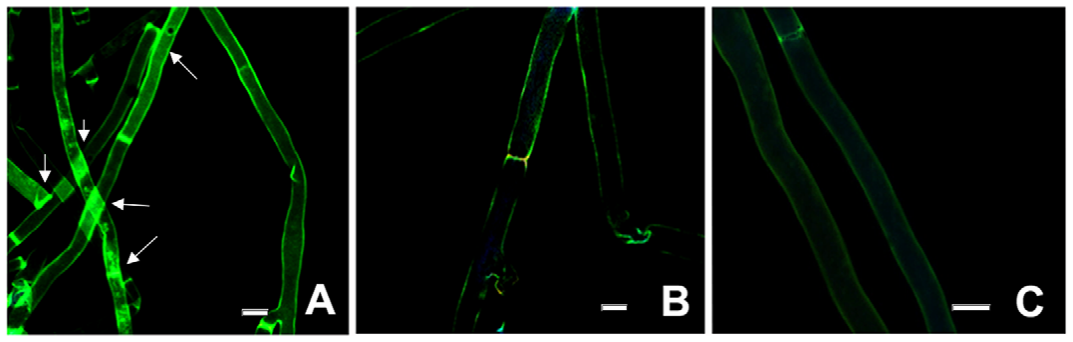
Immunolocalization of mASAL in *R. solani* hyphae. (A) Strong signals appeared in the hyphae of mASAL treated *R. solani*. In contrast, in (B), no signals were generated in the hyphae treated with ASAL. (C) Negative control (PBS buffer, pH 7.4). White arrows indicated presence of signals. Bars represent 10 µm.

Fluorescent microscopic analysis of *R. solani* hyphae revealed direct binding of mASAL to the fungal membrane when probed with a FITC tagged antibody (**Figure 9A**). The presence of high-intensity green fluorescence throughout the mycelium of *R. solani* is evidence of penetration of mASAL through the fungal external structural barrier. In contrast, absence of any signal indicated the exclusion of ASAL by the membranes of the hyphae (**Figure 9B**). Identical data were obtained in case of negative control sets (**Figure 9C**).

### Receptor identification through ligand blot assay and its characterization

**Figure 10:**
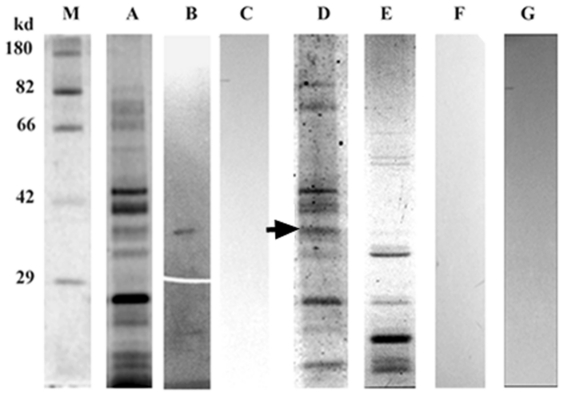
Identification and characterization of fungal receptor. (A) Staining of the subproteome of *R. solani* using a Sypro ruby stain. (B) An approximately 37 kDa putative receptor was identified in a ligand blot assay of the membrane subproteome of *R. solani* with mASAL when probed with an anti-ASAL antibody. (C) Ligand blot analysis of the subproteome with mASAL pre-saturated with excess α- D mannose and probed with an anti-ASAL antibody showed an absence of signal (D) Glycospecific-staining of subproteome indicated the glycoprotein nature of the putative receptor (arrowhead denoting an approximately 37-kDa receptor) (E) Staining of deglycosidase-treated subproteome by Sypro ruby stain (F) Ligand blot analysis of de-glycosylated subproteome probed with an anti-ASAL antibody showed no signal (G) A ligand blot assay of a subproteome without mASAL as a negative control (M) represents a standard Protein molecular weight marker.

Ligand blot analysis of the membrane subproteome of *R. solani* with mASAL demonstrated signal at the 37-kDa region when probed with an anti-ASAL antibody (**Figure 10B**). Glycoprotein-specific staining of the subproteome showed band near the 37-kDa region (**Figure 10D**), clearly indicating the glycoprotein nature of the putative receptor. Furthermore, Sypro ruby staining of the subproteome detected all of the glycosylated and non-glycosylated proteins (**Figure 10A**). Ligand blot analysis of the de-glycosylated subproteome (**Figure 10E**) with mASAL probed with an anti-ASAL antibody showed an absence of any signal (**Figure 10F**). These data certainly indicated that the interaction of mASAL with the receptor protein is a lectin glycoprotein-like interaction, as the ligand binding ability of the putative receptor was abolished after de-glycosylation. Mannose inhibition was carried out by mASAL pre-saturated with excess α –D mannose and subsequent ligand blot assays were performed with subproteome and pre-saturated mASAL probed with an anti-ASAL antibody. Absence of signal (**Figure 10C**) indicated that the interaction of mASAL with the putative receptor was mannose-mediated. Ligand blot assay of membrane subproteome without mASAL (**Figure 10G**) was designated as a negative control.

## Discussion

### The five-residue motif (-DNSNN-) plays a key role in switching the dimer to stable monomer

Comparative analyses of amino acid sequences, structures and protein oligomerization of monocot mannose binding lectins have been studied recently [30]. This super family is quite heterogeneous in its quaternary organization, regardless of the high degree of sequence conservation and structural similarity. The present study demonstrates that it is possible to radically change the specificity and functionality of ASAL into mASAL through a rational protein engineering approach. Despite being a monomer, mASAL resembles the general fold of known monocot mannose binding lectins, the β-prism II fold, which has been established since the initial availability of the reported structure of GNA [31]. During mutagenesis, the mannose binding motif and the aromatic residues Trp 41, Trp 74, and Trp 103 were kept unchanged so that the structural fold was maintained as it is in the dimeric ASAL. The CD spectroscopic analyses established the similar conformations for dimeric ASAL as well as the monomeric mutant ASAL (**Figure 4** **C, D**). The incorporation of the five-residue motif (belonging to 3∶5 β-hairpins with a β-bulge inside forming a β-hairpin) contributed to the C-terminal self-assembly rather than the C-terminal exchange mode essential for dimerization. Such rearrangement of residues causes a large decrease in the hydrophobic surface at the interface. Therefore, the flanking 12^th^ β-strand is folded back towards the axis of the molecule, forming a homogeneous β-sheet. Due to the presence of the 3∶5 β-hairpin and the conformational shift of the 12^th^ β-strand in the mutated lectin, the monomeric structure is stabilized.

Though the exact mechanism of ligand binding was not discovered in our study, some binding features can be anticipated by comparison with other homologous sequences. The three putative mannose binding sites served by the stretch QXDXNXVXY in each monomer indicate a similar binding mode, and the conserved side chains of the binding pocket of the mutated lectin coincide well with those in GNA. This confirms its strong preference for the axial hydroxyl group at position 2 in the ligand, which is a common property of other lectins. However, the change adjacent to subdomain I may lead to variation in the size of the binding pocket, suggesting the possibility of binding ligands other than high mannose oligosaccharides. It is quite interesting that, although the geometries of the binding sites of GNA and ASAL are similar, their preferences for complex glycans may vary considerably.

### Quaternary association of garlic lectin strongly correlates with its functional activity

It has been well documented in studies of other lectins that variability in quaternary structure is in some way related to diverse ligand preferences. Some tetrameric lectins, such as GNA and the *Narscissus* lectin (NPL) display an inhibitory activity against retroviruses resulting from their strong affinity towards gp120, the major glycoprotein of human immunodeficiency virus. In contrast, garlic lectin, a dimer, does not. The structure of the snowdrop lectin complex with its branched mannopentose revealed two distinct binding modes. As evidenced from mannose binding experiments, it can be suggested that there is a distinct difference in the binding affinity of ASAL and mASAL towards molecular mannose. Because monomeric molecules lack contacts from neighboring subunits, it is hard to believe that monomers bind polysaccharides with complex branching like that of oligomers. This indicates that mASAL possesses a distinct preference for its ligands, which is different from that of oligomers.

The physiological role of monocot mannose binding lectins remains poorly understood thus far. The evidence in recent years has suggested that these lectins serve as devices for plant defense systems against the damage caused by insect pests. For example, ASAL has been established to impart beneficial effects against sucking-type insect pests when they are fed with artificial diets supplemented with ASAL in pure form or in an *in vivo* condition in transgenic plants expressing lectins [32]–[34]. On the contrary, mASAL has been found to portray an antifungal property against a number of pathogenic fungi harmful to crop plants. At the same time, in the case of mASAL, the hemagglutination property of lectin was lost and most likely a smaller number of carbohydrate binding sites in monomeric mASAL causes a loss of the multivalency essential for agglutination [12].

### Possible evolutionary implications of stable monomeric ASAL

Plant lectins play an important role in defense, as they have the unique proficiency to bind the carbohydrate part of glycoproteins and glycolipids. As we have previously discussed, the monocot mannose binding lectin superfamily includes lectins that have similar sequences but diverse functions. Recently, new lectin genes have been identified as being responsible for biotic and abiotic stress-related developmental processes [35]. Previously, Liu et al. 2005 [36] isolated and studied a monomeric form of mannose binding protein from orchid, gastrodianin, which exhibited antifungal activity that seems to exist as an evolved feature. In the present investigation, the stable monomeric form of ASAL was obtained by site-directed mutagenesis that obtained an antifungal property. Biochemical, bioinformatic and functional analyses provide a deeper insight into the evolutionary aspect of mannose binding protein mediated defense mechanisms in plants. Consequently, it can be hypothesized that monocot mannose binding lectins do not represent a monophylogenetic origin and that nature has evolved these defensive proteins of different quaternary states as adaptations to variable environmental conditions.

### An insight into the mode of action of mASAL towards fungal pathogen

In recent past, several plant proteins have been identified as antifungal proteins and have been applied biotechnologically to protect various crop plants; these include chitinases [37], hevein type proteins [38]-[39], plant defensins [40], GAFPs [36], etc. On the basis of sequence homology and oligomerization level, mASAL is very close to GAFP. The inhibition profile of mASAL is interesting and promising from the perspective of plant biotechnology. The results presented in Figure 8 demonstrate that in comparison to ASAL, mASAL alters the fungal membrane permeability. Indeed, one could hypothesize that this alteration depends on its lectin activity, because as determined via the binding assay, mASAL was found to bind mannose efficiently. To test this hypothesis, we performed a mannose inhibition assay (Figure 8) and a ligand blot assay (Figure 10). The data obtained from the propidium iodide uptake assay are consistent with the data from the ligand blot assay. Both approaches strongly suggest that mASAL binds to the glycoprotein component or mannans of the fungal cell wall and thereby alters the normal cross linking activities during cell wall formation [41]-[42], severely affecting the growth and pathogenicity of the fungus. Only the monomeric form is able to display such an effect due to its smaller size, which allows it to explore the glycoprotein component of the cell wall because the size exclusion for a typical antifungal protein is limited to approximately 15–20 kDa [36]. It is also evident from the bioassay data that the fusion protein MBP-mASAL (54 kDa) shows no inhibitory activity against any of the tested fungi, whereas the purified mASAL is detrimental to fungal pathogens. Finally, as determined by immunolocalization assay (**Figure 9**), we can convincingly provide evidence that in comparison to ASAL, mASAL is more successful in penetrating the fungal membrane. Additionally, a comprehensive understanding is required to identify and characterize the fungal membrane protein receptor through a functional proteomic approach in order to determine the actual mechanism of the antifungal activity.

### Conclusion

In conclusion, our study demonstrated that it is possible to radically change the oligomerization level of ASAL by the insertion and replacement of five residues (-DNSNN-) resulting in a stable monomeric protein variant.

Interestingly, mASAL gains a potent antifungal activity against a number of important fungal pathogens. Presumably, this altered biological activity with the altered oligomeric status provides us clues with which to hypothesize that perhaps it is the evolutionary pressure (under which plants have to survive) that might have led to the evolution of the varied quaternary organizations of a protein. Certainly, this study has implications towards an understanding of the evolutionary relationship among monocot mannose binding lectins. Further in-depth studies are required to resolve several questions regarding a range of quaternary organizations of this fascinating lectin super family.

The present study provides the possibility of extending the arena of utility of mannose binding proteins in the sphere of plant biotechnology. In conclusion, the antifungal activity of this protein is promising enough to merit further investigation of its potential in biotechnology approaches to increase fungal resistance in agronomically important crop species.

## Supporting Information

Figure S1 **Propidium iodide uptake assay on** ***Xanthomonas oryzae*** **Bxo43 bacteria.** (A) Fluorescent microscopic images of heat killed bacteria stained with PI, used as a positive control. ‘Heat killed’ indicates 10 min treatment at 65°C. (B) Fluorescent microscopic images of mASAL-treated (4 µg) bacteria stained with PI. (C) Fluorescent microscopic images of ASAL-treated (4 µg) bacteria stained with PI. (D) Fluorescent microscopic images of untreated bacteria stained with PI, used as a negative control. Scale bar: 10 µm.
(TIF)
