# Supplementary figures and images for: Functional Alteration of a Dimeric Insecticidal Lectin to a Monomeric Antifungal Protein Correlated to Its Oligomeric Status
Source: PLoS One. 2011 Apr 7;6(4):e18593. doi: 10.1371/journal.pone.0018593 (PMC3072408; doi:10.1371/journal.pone.0018593)

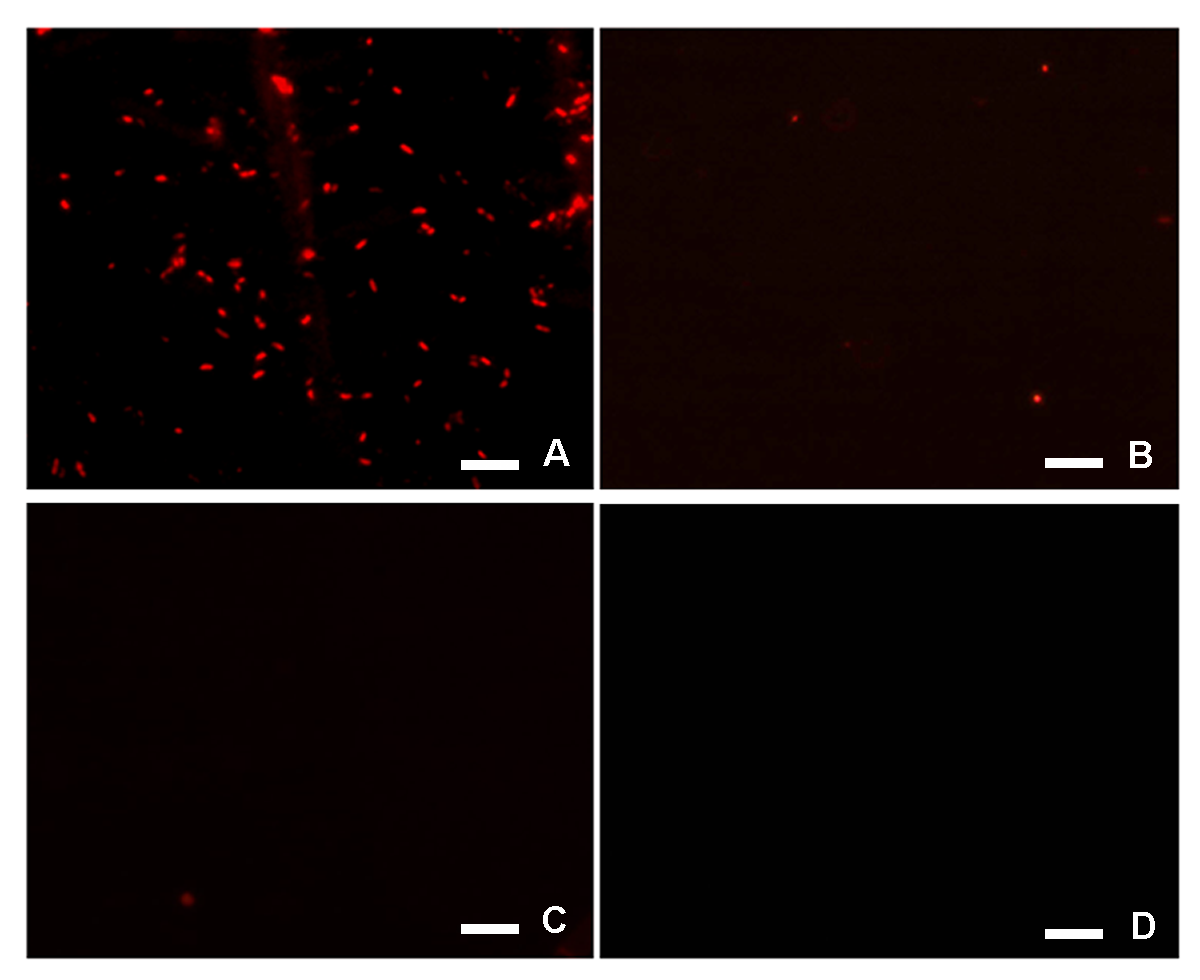

Supplement: Figure S1 — Propidium iodide uptake assay on Xanthomonas oryzae Bxo43 bacteria. (A) Fluorescent microscopic images of heat killed bacteria stained with PI, used as a positive control. ‘Heat killed’ indicates 10 min treatment at 65°C. (B) Fluorescent microscopic images of mASAL-treated (4 µg) bacteria stained with PI. (C) Fluorescent microscopic images of ASAL-treated (4 µg) bacteria stained with PI. (D) Fluorescent microscopic images of untreated bacteria stained with PI, used as a negative control. Scale bar: 10 µm. (TIF) [file pone.0018593.s001.tif]
